# Allergic history and responses to immunotherapy in individuals with recurrent or metastatic head and neck squamous cell carcinoma

**DOI:** 10.3389/fonc.2025.1486583

**Published:** 2025-03-25

**Authors:** Yannan Wang, Zhonghui Ma, Qizhe Zheng, Yinglin Chu, Yunshuang Hu, Fei Liu

**Affiliations:** Department of Stomatology, The First Affiliated Hospital of Zhengzhou University, Zhengzhou, China

**Keywords:** head and neck squamous cell carcinoma, immunotherapy, allergy, smoking, clinical response

## Abstract

**Objective:**

To elucidate the association between allergy history and response to immune checkpoint inhibition (ICI) in recurrent or metastatic head and neck squamous cell carcinoma (RM/HNSCC).

**Methods:**

Patients receiving ICI treatment for RM/HNSCC were retrospectively enrolled and classified into two groups based on their previous allergy history. The primary outcome variable assessed was the response to ICI.

**Results:**

A total of 157 patients were included, of whom 27 reported a history of allergies. In multivariate analysis, patients with allergies exhibited an odds ratio of 2.78 [95% confidence interval: 1.54-5.99], significantly surpassing that of the non-allergic group. Other independent predictors of ICI benefit included current smoking status and the primary tumor site being in the oropharynx or hypopharynx. Neither progression-free survival nor overall survival was adversely affected by prior allergy history or smoking status or HPV status or PD-L1 expression.

**Conclusion:**

A prior history of allergies is associated with an enhanced response to immunotherapy in patients with RM/HNSCC.

## Introduction

Head and neck squamous cell carcinoma (HNSCC) is the sixth most common malignancy among all solid cancers, involving more than 850,000 cases annually in the world (1). The immutable risk factors for HNSCC encompass smoking, alcohol consumption, betel nut chewing, and human papillomavirus (HPV) infection. Additionally, the immune system’s response has emerged as a novel risk factor for HNSCC. Numerous studies have probed the relationship between allergic manifestations of the immune response and cancer risk, including pancreatic and prostate cancers (2). Individuals with pronounced atopic tendencies exhibit a heightened propensity to develop allergies, attributable to their exaggerated immune responses to specific environmental antigens. These individuals possess a genetic predisposition to produce elevated levels of immunoglobulin E (IgE) against common allergens. The cross-linking of IgE on the surfaces of particular leukocytes can precipitate allergic disorders such as allergic rhinitis, atopic dermatitis, asthma, and other allergic reactions, including food allergies (3).

The deleterious impact of allergies on cancer development has been elucidated through various hypotheses, with the “prevention hypothesis” and the “immune surveillance hypothesis” being the most prominent. According to the immune surveillance hypothesis, the incidence of allergies is attributed to an overactive immune system that efficiently eradicates malignant cells, thereby diminishing the risk of cancer. Conversely, the prevention hypothesis posits that allergic symptoms are instigated by the swift action of toxins, microorganisms, and environmental particulates that carry or contain carcinogens. Furthermore, allergic symptoms can deter individuals from exposure to perilous environments (4). In the case of glioma and pancreatic cancer, both of which are associated with well understood environmental risk factors, evidence has consistently demonstrated that allergies exert a protective effect (5), but role of allergies on HNSCC seem less reliable.

Even following curative interventions, a significant proportion—30% or more—of patients with HNSCC are susceptible to recurrence. In this context, immune checkpoint inhibition (ICI) has emerged as a beacon of hope, representing a promising therapeutic avenue. Markers such as PD-L1, PD-1, CTLA-4, p53, and tumor mutational burden have been identified as potential factors that may augment the efficacy of treatment, thereby enhancing clinical outcomes for affected patients (6).

While the interconnection between allergic conditions and the incidence of HNSCC has been extensively investigated (7, 8), the interplay between allergies and the efficacy of immunotherapy in patients with HNSCC remains an enigma. The objective of this study is to elucidate the association between the allergy history reported by patients with recurrent or metastatic HNSCC and their subsequent response to ICIs.

## Patients and methods

### Ethical considerations

This study was approved by the Zhengzhou University Institutional Research Committee. All participants provided written informed consent at initial treatment for medical research. All procedures involving human participants were conducted according to the principles of the Declaration of Helsinki.

### Study design

To achieve our objective, we conducted a retrospective review of patients treated with isolated PD-1/PD-L1 or CTLA-4 inhibitors—including pembrolizumab, nivolumab, ipilimumab, or durvalumab—for unresectable recurrent or metastatic HNSCC between January 1, 2020, and December 2023. Patients were excluded from the analysis if they met any of the following criteria: individuals with primary HNSCC; patients who had succumbed to their illness or were lost to follow-up prior to receiving post-treatment imaging to assess clinical response; individuals under the age of 18; and those with an unknown primary tumor. Baseline data pertinent to immunotherapy encompassed the following: treatment commencement date, patient age, gender, smoking status, Eastern Cooperative Oncology Group (ECOG) performance score, self-reported allergy history, tumor status determination concerning HPV through p16 analysis via immunohistochemical examination or *in situ* hybridization, and the primary tumor location, prior treatment.

### Variable definition

Histopathological slides were meticulously examined by at least two experienced pathologists specializing in head and neck pathology. Current smokers were defined as individuals who consistently consumed a minimum of 20 cigarettes daily for at least one year, without significant cessation periods. Former smokers were classified as those who had quit smoking for no less than two years, while never smokers referred to individuals who had never engaged in smoking (9). The combined positive score (CPS) was defined as the number of PD-L1-staining cells (tumor cells, lymphocytes, and macrophages) divided by the total number of viable tumor cells, then multiplied by 100 based on the 22C3 platform (10). Data on allergy history were obtained via self-reported information that included drug allergies, food allergies, and environmental allergies.

### Statistical analysis

The primary outcome variable was the response to immunotherapy, determined based on the RECIST guidelines. Patients were classified as having a clinical response to immunotherapy if they exhibited either a complete or partial response; conversely, patients with stable or progressive disease following ICI treatment were categorized as non-responders. Throughout the efficacy assessment phase, ICI were administered continuously until disease progression was observed or until such time as the toxicity became intolerable. The efficacy was determined in accordance with the most favorable response recorded over the entire treatment period, as ascertained through CT and MRI scans, which were conducted at intervals of 3 to 6 months (11). Secondary outcome variables included progression-free survival (PFS) and overall survival (OS).

In the assessment of the primary outcome, potential influencing factors were initially scrutinized through the application of the Chi-square test. Subsequently, those variables that demonstrated statistical significance were subjected to further examination via multivariable logistic regression analysis, with the results presented in terms of odds ratios (ORs) and 95% confidence intervals (CIs). For the secondary outcome, a comparative analysis of clinicopathologic variables between the allergy and non-allergy groups was conducted using the Chi-square test. Following this, patients were meticulously matched in a 1:1 ratio across the two groups by calculating the propensity scores for those variables that were found to be statistically significant. The impact of allergy history on PFS and OS was then analyzed within the matched cohort employing the Kaplan-Meier method. All statistical analyses were performed utilizing R version 3.4.3, with a p-value threshold of less than 0.05 deemed indicative of statistical significance.

## Results

### Baseline data

A total of 157 patients were enrolled in the study, comprising 123 males and 34 females, with a mean age of 60 ± 11 years. Body Mass Index (BMI) was classified as follows: 69 patients (43.9%) had a BMI of less than 18.5, 72 patients (45.9%) fell within the range of 18.5-23.9, and 16 patients (10.2%) had a BMI of 24.0 or greater. Smoking status was recorded, revealing that 43 patients (27.4%) were current smokers, 96 patients (61.1%) were former smokers, and 18 patients (11.5%) had never smoked. The primary tumor sites included the oral cavity in 37 patients (23.6%), the oropharynx in 46 patients (29.3%), the larynx in 22 patients (14.0%), and the hypopharynx in 52 patients (33.1%). Among the cohort, 10 patients tested positive for HPV. Notably, 28 patients (17.8%) exhibited a CPS of less than 1, while 62 patients (39.5%) had a CPS exceeding 20.

Of the 157 patients, 27 (17.2%) had a documented history of allergies, which appeared to correlate significantly with smoking status (p=0.029); however, there were no notable differences in demographic or pathological variables between the allergy and no-allergy groups (all p>0.05, as detailed in **Table 1**). Subsequently, the smoking status factor was incorporated into propensity score calculations with a 1:1 ratio, resulting in the enrollment of 54 patients (27 from each group) for survival analysis (**Table 2**).

**Table 1:** Baseline data of the enrolled patients.

Variable	Overall (n=157)	Allergy group (n=27)	No-allergy group (n=130)	p*
Age				
≤60	60 (38.2%)	10 (37.0%)	50 (38.5%)	
>60	97 (61.8%)	17 (63.0%)	80 (61.5%)	0.890
Sex				
Male	123 (78.3%)	21 (77.8%)	102 (78.5%)	
Female	34 (21.7%)	6 (22.2%)	28 (21.5%)	0.937
BMI				
~18.4	69 (43.9%)	10 (37.0%)	59 (45.4%)	
18.5-23.9	72 (45.9%)	12 (44.4%)	60 (46.2%)	
24.0+	16 (10.2%)	5 (18.5%)	11 (8.5%)	0.277
Smoking				
Current	43 (27.4%)	5 (18.5%)	38 (29.2%)	
Former	96 (61.1%)	15 (55.6%)	81 (62.3%)	
Never	18 (11.5%)	7 (25.9%)	11 (8.5%)	0.029
Site				
Oral	37 (23.6%)	7 (25.9%)	30 (23.1%)	
Oropharynx	46 (29.3%)	8 (29.6%)	38 (29.2%)	
Larynx	22 (14.0%)	4 (14.8%)	18 (13.8%)	
Hypopharynx	52 (33.1%)	8 (29.6%)	44 (33.8%)	0.990
HPV status				
Positive	10 (6.4%)	2 (7.4%)	8 (6.2%)	
Negative	147 (93.6%)	25 (92.6%)	122 (93.8%)	0.808
CPS^				
~0.9	28 (17.8%)	4 (14.8%)	24 (18.5%)	
1-20	67 (42.7%)	11 (40.7%)	56 (43.1%)	
21+	62 (39.5%)	12 (44.4%)	50 (38.5%)	0.886
ECOG performance score^#^				
0-1	130 (82.8%)	22 (81.4%)	108 (83.1%)	
2	27 (17.2%)	5 (18.6%)	22 (16.9%)	1.000
Prior treatment^!^				
S	19 (12.1%)	3 (11.1%)	16 (12.3%)	
CRT	36 (22.9%)	8 (29.6%)	28 (21.5%)	
S+R	48 (30.6%)	8 (29.6%)	40 (30.8%)	
S+CRT	54 (34.4%)	8 (29.6%)	46 (35.4%)	0.833
Immunotherapy type				
Pembrolizumab	127 (80.9%)	20 (74.1%)	107 (82.3%)	
Nivolumab	17 (10.8%)	4 (14.8%)	13 (10.0%)	
Ipilimumab	7 (4.5%)	2 (7.4%)	5 (3.8%)	
Durvalumab	6 (3.8%)	1 (3.7%)	5 (3.8%)	0.814

*comparison between allergy and no-allergy groups.

^CPS, combined positive score.

#ECOG, Eastern Cooperative Oncology Group.

!S, surgery; CRT, chemoradiotherapy; R, radiotherapy.

**Table 2:** Baseline data of the enrolled patients after propensity Score Matching.

Variable	Allergy group (n=27)	No-allergy group (n=27)	p*
Age			
≤60	10 (37.0%)	11 (40.7%)	
>60	17 (63.0%)	16 (59.3%)	0.780
Sex			
Male	21 (77.8%)	20 (74.1%)	
Female	6 (22.2%)	7 (25.9%)	0.750
BMI			
~18.4	10 (37.0%)	12 (44.4%)	
18.5-23.9	12 (44.4%)	10 (37.0%)	
24.0+	5 (18.5%)	5 (18.5%)	0.834
Smoking			
Current	5 (18.5%)	5 (18.5%)	
Former	15 (55.6%)	15 (55.6%)	
Never	7 (25.9%)	7 (25.9%)	1.000
Site			
Oral	7 (25.9%)	7 (25.9%)	
Oropharynx	8 (29.6%)	8 (29.6%)	
Larynx	4 (14.8%)	4 (14.8%)	
Hypopharynx	8 (29.6%)	8 (29.6%)	1.000
HPV status			
Positive	2 (7.4%)	2 (7.4%)	
Negative	25 (92.6%)	25 (92.6%)	1.000
CPS^			
~0.9	4 (14.8%)	4 (14.8%)	
1-20	11 (40.7%)	11 (40.7%)	
21+	12 (44.4%)	12 (44.4%)	1.000
ECOG performance score^#^			
0-1	22 (81.4%)	23 (85.2%)	
2	5 (18.6%)	4 (4.8%)	1.000
Prior treatment^!^			
S	3 (11.1%)	2 (7.4%)	
CRT	8 (29.6%)	9 (33.3%)	
S+R	8 (29.6%)	7 (25.9%)	
S+CRT	8 (29.6%)	9 (33.3%)	1.000
Immunotherapy type			
Pembrolizumab	20 (74.1%)	22 (81.5%)	
Nivolumab	4 (14.8%)	3 (11.1%)	
Ipilimumab	2 (7.4%)	1 (3.7%)	
Durvalumab	1 (3.7%)	1 (3.7%)	0.884

*comparison between allergy and no-allergy groups.

^CPS, combined positive score.

#ECOG, Eastern Cooperative Oncology Group.

! S, surgery; CRT, chemoradiotherapy; R, radiotherapy.

### Predictors for immunotherapy response

A complete or partial response to immunotherapy was achieved in 38 patients (24.2%). Among the 27 patients with a history of allergies, a clinical response was observed in 37.0% of this population, which was significantly higher than that of the no-allergy group (p=0.014). The clinical response rates were recorded as 34.8% for current smokers, 22.9% for former smokers, and a mere 5.6% for never smokers; this variation was statistically significant (p=0.046). Oral cancer predicted a clinical response rate of 13.5%, while the frequency was 21.7% for oropharyngeal cancer and 18.2% for laryngeal cancer, all exhibiting significantly lower response rates compared to hypopharyngeal cancer, which had a response rate of 36.5% (p=0.064). Furthermore, 50% of HPV-positive patients achieved clinical response, suggesting a trend towards higher responsiveness compared to the negative cohort (p=0.063). All these factors were further scrutinized in multivariable analysis. Clinical response showed no significant association with other variables (**Table 3**).

**Table 3:** Association between clinicopathologic variables and immunotherapy response.

Variable	Complete or partial response (n=38)	Progressive or stable disease (n=119)	p
Age			
≤60	14 (36.8%)	46 (38.6%)	
>60	24 (63.2%)	73 (61.4%)	0.841
Sex			
Male	32 (84.2%)	91 (76.5%)	
Female	6 (15.8%)	28 (23.5%)	0.313
BMI			
~18.4	16 (42.1%)	53 (44.5%)	
18.5-23.9	18 (47.4%)	54 (45.4%)	
24.0+	4 (10.5%)	12 (10.1%)	0.963
Smoking			
Current	15 (39.5%)	28 (23.5%)	
Former	22 (57.9%)	74 (62.2%)	
Never	1 (2.6%)	17 (14.3%)	0.046
Site			
Oral	5 (13.2%)	32 (26.9%)	
Oropharynx	10 (26.3%)	36 (30.2%)	
Larynx	4 (10.5%)	18 (15.1%)	
Hypopharynx	19 (50.0%)	33 (27.7%)	0.064
HPV status			
Positive	5 (13.2%)	5 (4.2%)	
Negative	33 (86.8%)	114 (95.8%)	0.063
CPS^			
~0.9	4 (10.5%)	24 (20.2%)	
1-20	15 (39.5%)	52 (43.7%)	
21+	19 (50.0%)	43 (36.1%)	0.220
Allergy history			
Yes	11 (28.9%)	16 (12.3%)	
No	27 (71.1%)	114 (87.3%)	0.014
ECOG performance score^#^			
0-1	31 (81.6%)	99 (83.2%)	
2	7 (18.4%)	20 (16.8%)	0.818
Prior treatment^!^			
S	4 (10.5%)	15 (12.6%)	
CRT	10 (26.3%)	26 (21.8%)	
S+R	11 (28.9%)	37 (31.1%)	
S+CRT	13 (34.2%)	41 (34.5%)	0.953
Immunotherapy type			
Pembrolizumab	31 (81.6%)	96 (80.7%)	
Nivolumab	3 (7.9%)	14 (11.8%)	
Ipilimumab	2 (5.3%)	5 (4.2%)	
Durvalumab	2 (5.3%)	4 (3.4%)	0.836

^CPS, combined positive score

#ECOG, Eastern Cooperative Oncology Group.

!S, surgery; CRT, chemoradiotherapy; R, radiotherapy.

In logistic regression analysis, patients with allergies demonstrated an odds ratio (OR) of 2.78 [95%CI: 1.54-5.99], which was significantly elevated compared to the no-allergy group (p<0.001). When compared to never smokers, former smokers exhibited a comparable OR (95% CI: 0.95-2.87), whereas current smokers demonstrated a significantly increased OR of 2.01 (95% CI: 1.36-3.00). Both oral and laryngeal cancers displayed similar ORs, while oropharyngeal and hypopharyngeal cancers were more likely to benefit from immunotherapy, with ORs of 1.47 (95% CI: 1.12-2.36) and 2.98 (95% CI: 1.14-4.87), respectively. HPV status did not significantly influence the likelihood of achieving a clinical response (**Tables 4**).

**Table 4:** Logistic analysis of the association between clinical pathologic variables and immunotherapy response.

Variable	OR [95%CI]	p
Smoking		
Never	Reference	
Former	1.85 [0.95-2.87]	0.079
Current	2.01 [1.36-3.00]	0.011
Site		
Oral	Reference	
Oropharynx	1.47 [1.12-2.36]	0.024
Larynx	1.37 [0.78-1.98]	0.138
Hypopharynx	2.98 [1.14-4.87]	<0.001
HPV status		
Positive	Reference	
Negative	1.28 [0.47-1.75]	0.391
Allergy history		
No	Reference	
Yes	2.78 [1.54-5.99]	<0.001

### PFS and OS

**Figure 1:**
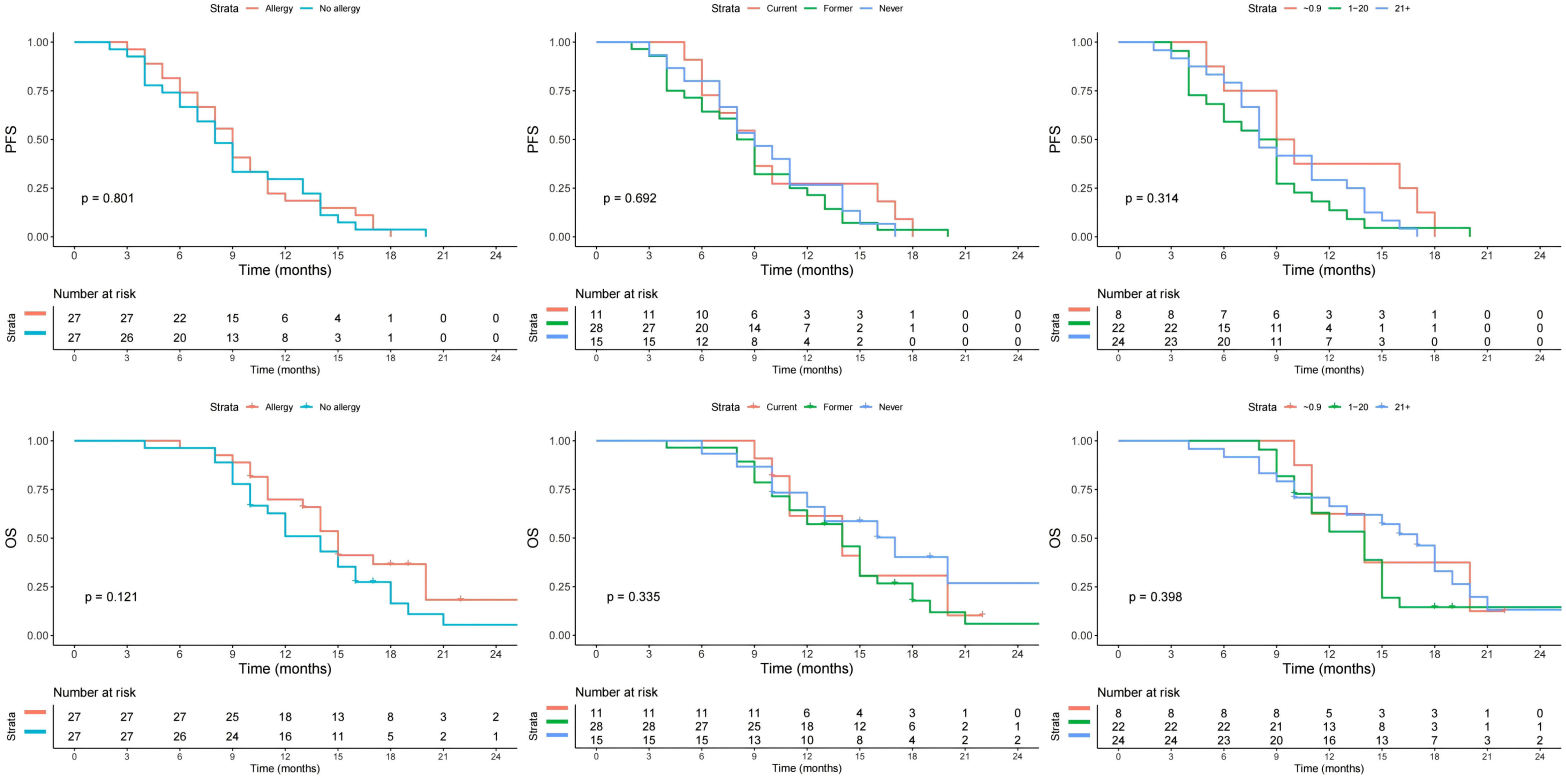
Progression free survival (PFS) and overall survival (OS) in patients with different allergy status, smoking status, and CPS.

**Figure 2:**
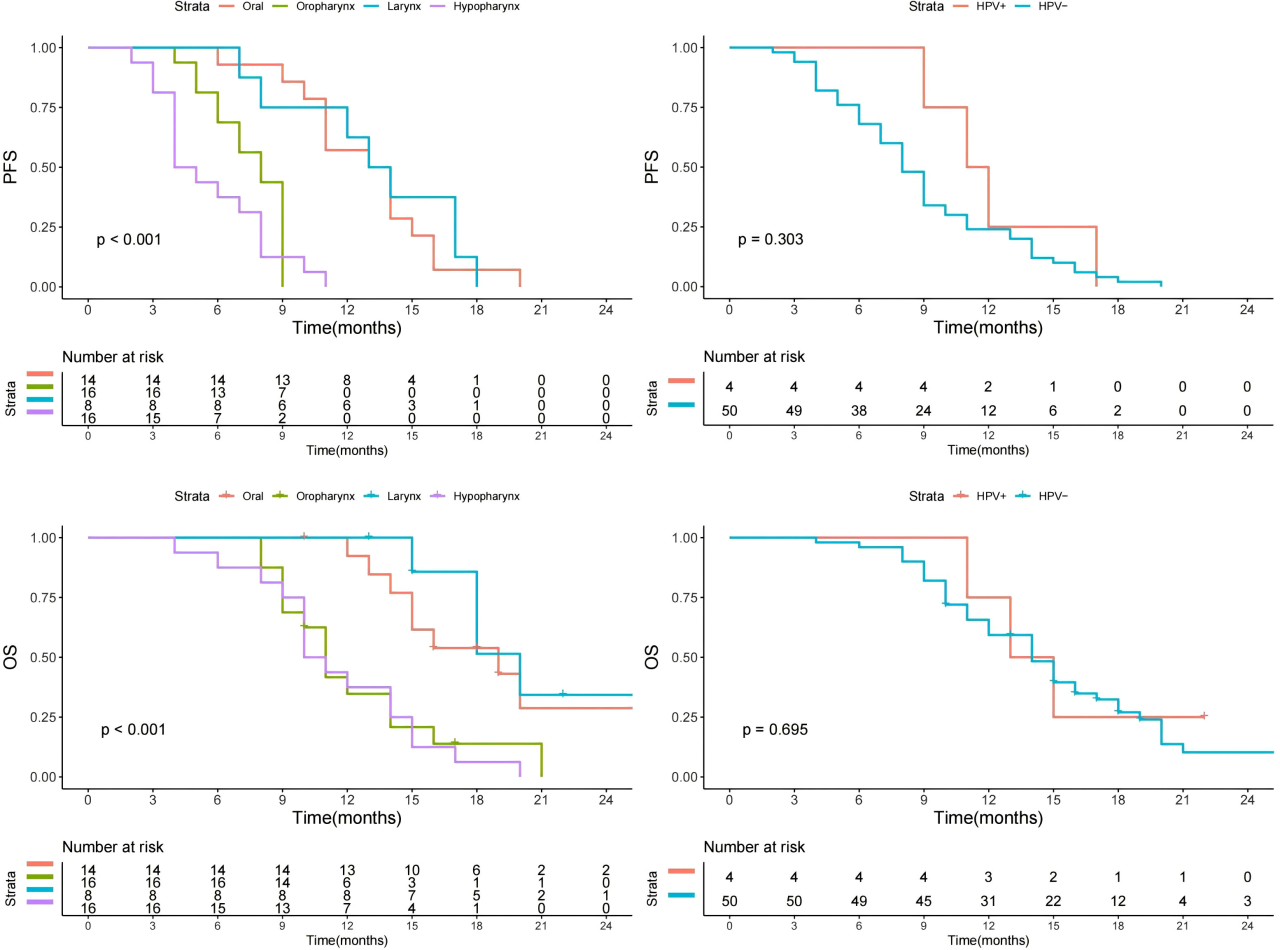
Progression free survival (PFS) and overall survival (OS) in patients with different HPV status, and primary sites.

During the median follow-up period of 14 months (range: 4-40), all patients experienced disease progression, leading to the death of 132 individuals. Following the implementation of propensity score matching, no statistically significant disparities were observed in either PFS (p=0.801) or OS (p=0.121) between patients with and those without a history of allergies. Additionally, neither smoking status nor CPS or HPV status exerted a discernible influence on PFS or OS. However, the primary anatomical sites demonstrated a significant impact on both PFS and OS, with tumors originating in the hypopharynx or oropharynx portending the most dire prognostic outcomes, as illustrated in **Figures 1** and **2**.

In the whole cohort, the sole independent prognostic factor identified was the primary tumor site; when compared to patients afflicted with oral carcinoma, those presenting with squamous cell carcinoma of the oropharynx and hypopharynx demonstrated a markedly inferior PFS and OS (**Tables 5**, **6**).

**Table 5:** Univariate analysis of factors influencing progression-free survival (PFS) and overall survival (OS) in the whole cohort.

Variable	PFS	OS
Age (>60 vs ≤60)	0.174	0.290
Sex (Male vs female)	0.300	0.467
BMI (24.0+ vs 18.5-23.9 vs ~18.4)	0.190	0.223
Smoking (Current vs Former vs Never)	0.548	0.417
Site (Hypopharynx vs Larynx vs Oropharynx vs Oral)	<0.001	<0.001
HPV status (Negative vs positive)	0.013	0.022
CPS^ (21+ vs 1-20 vs ~0.9)	0.245	0.307
Allergy history (Yes vs no)	0.742	0.267
ECOG performance score^#^ (2 vs 0-1)	0.428	0.175
Prior treatment^!^ (S+CRT vs S+R vs CRT vs S)	0.674	0.557

^CPS, combined positive score

#ECOG, Eastern Cooperative Oncology Group.

! S, surgery; CRT, chemoradiotherapy; R, radiotherapy.

**Table 6:** Multivariable analysis of factors influencing progression-free survival (PFS) and overall survival (OS) in the whole cohort.

Variable	PFS		OS	
	HR [95%CI]	p	HR [95%CI]	p
Site				
Oral	ref		ref	
Oropharynx	2.21 [1.13-5.28]	0.003	1.78 [1.08-3.43]	0.009
Larynx	1.40 [0.54-3.31]	0.336	1.36 [0.52-3.21]	0.289
Hypopharynx	3.33 [1.54-7.78]	<0.001	3.55 [1.67-8.45]	<0.001
HPV status				
Positive	ref		ref	
Negative	1.40 [0.64-5.17]	0.276	1.58 [0.43-6.11]	0.327

## Discussion

This study reveals a significant correlation between allergy history and the response to ICI in patients with recurrent or metastatic HNSCC. To the best of the author’s knowledge, this research represents a pioneering effort in the literature to explore this specific association. Traditional biomarkers for immunotherapy, such as tumor mutation burden and PD-L1 expression in lung tumors, have been established; however, the prognostic value and applicability of PD-L1 in HNSCC remain unproven. Moreover, assessing mutation burden is often impeded by substantial financial costs and prolonged turnaround times for results. In contrast, a patient’s allergy history is readily accessible and easily ascertainable during virtually any clinical interaction. This accessibility enhances the practicality of utilizing allergy history as a predictive tool for immunotherapy response in patients with recurrent or metastatic HNSCC, thereby facilitating informed therapeutic decisions.

Patients exhibiting a clinically beneficial response to immunotherapy were likely to demonstrate a history of allergies, but survival analysis suggests that a history of allergies did not confer a protective effect against disease progression and mortality in patients undergoing ICI treatment for recurrent or metastatic HNSCC. Potential explanations may be multifaceted, encompassing our relatively modest sample size, the inherently aggressive characteristics of recurrent or metastatic HNSCC, as well as other deleterious pathologic attributes. This study substantiates the notion that the response to immunotherapy is influenced by various factors, including tumor mutation burden, PD-L1 expression, inflammatory gene expression profiles, tumor grading, and pre-treatment nutritional status (12). Each of these variables may embody distinct yet complementary mechanisms that enhance the efficacy of immunotherapy in patients with recurrent or metastatic HNSCC.

Allergies and atopic symptoms are characterized by a systemic enhancement of Th2 immunity, which can potentially fortify antitumor immunity and mitigate cancer risk in allergic individuals (13). This systemic predisposition towards Th2 immunity may also facilitate improved responses to ICI in the context of allergic reactions observed in this study. Moreover, recent advancements have yielded increasing evidence supporting the pivotal role of IL-9 production by CD4+ T helper cells (Th9) in mediating anti-tumor immunity, particularly in melanoma (14). A study involving 46 patients found early elevations of Th9 cell counts in melanoma patients treated with nivolumab correlated with clinical improvement. Additionally, both IL-9 and IL-31-producing CD4+ T cells were upregulated in patients with respiratory, food, and skin allergies. It remains to be determined whether patients with allergies experience a more pronounced increase in Th9 cell counts and IL-9 following ICI treatment (15). Future research exploring comparative changes in cytokine levels, immunoglobulin levels, and overall immunity in patients with a history of allergies versus their non-allergic counterparts undergoing ICI treatment for metastatic HNSCC may elucidate the inflammatory mechanisms underlying the enhanced ICI response observed in allergic individuals in this study. Furthermore, investigations targeting melanoma patients undergoing immune checkpoint inhibition have indicated that those who concurrently utilized antihistamines exhibited significantly lower mortality rates compared to those who did not (16). Given that antihistamines are often prescribed to manage allergic symptoms, it is imperative that further studies assess the concurrent use of antihistamines and ICI treatment in both allergic and non-allergic patients to ascertain their combined effects on therapeutic outcomes.

Smoking is a well-established carcinogenic factor for HNSCC and also exerts a significant impact on allergy (17). Prior studies have analyzed the association between smoking and immunotherapy. In a cohort of 962 non-small cell lung cancer patients treated with immunotherapy, a matched analysis within the pembrolizumab group revealed that never smokers had a significantly shorter PFS and a non-significant trend towards reduced OS. Conversely, never smokers demonstrated a significantly longer PFS and OS compared to current and former smokers within the matched chemotherapy cohort. Pooled multivariable analysis confirmed that the interaction term between smoking status and treatment modality was statistically significant concerning objective response rate (p = 0.0074), PFS (p = 0.0001), and OS (p = 0.0020), underscoring the markedly different impacts of smoking status across the two cohorts (18). A recent review (19) evaluating the relationship between smoking status and the efficacy of PD-1/PD-L1 inhibitors compared with conventional agents analyzed 15 qualifying trials involving 9073 patients. Findings indicated that PD-1/PD-L1 inhibitors correlated with prolonged PFS and OS in current and former smokers but not in never smokers, regardless of cancer type, target of experimental agents, or treatment strategy. While the current study did not observe a significant impact of smoking status on survival, there was a notable association between clinical response and smoking. This finding is particularly intriguing; first, only unresectable recurrent or metastatic HNSCC patients were enrolled in the current study, which typically carries a poor prognosis and where only 24.2% of patients derive benefit from immunotherapy (20). Second, similar research, after adjusting for HPV status, revealed that patients with a history of allergies exhibited significantly decreased risk of disease progression and death with ICI compared to their non-allergic counterparts, a difference potentially explained by the former study not clarifying whether salvaged surgery was performed. Third, there existed discrepancies in tumor biology or the immune microenvironment between HNSCC and other malignant neoplasms.

The immune subterfuge and therapeutic resistance exhibited by HPV positive malignant neoplasms have ignited a keen interest in the application of established immunotherapeutic modalities to these carcinomas. ICI represent the most exhaustively evaluated strategy to date, having conducted the most extensive clinical investigation into HPV positive cancers within the realm of HNSCC. Numerous prior clinical trials did not impose restrictions on HPV status, thereby allowing the participation of HPV negative patients, which has complicated the interpretation of the outcomes. In the context of recurrent platinum-resistant HNSCC, the CHECKMATE-141 trial (21), a phase III investigation of monotherapy with nivolumab versus conventional therapy, demonstrated a significant enhancement in the OS rate with nivolumab (hazard ratio of 0.70, with a 1-year survival rate of 36% compared to 16%). The KEYNOTE-040 trial (22), a phase III study comparing pembrolizumab with standard care in the second-line setting of HNSCC, reported analogous enhancements in the OS rate for the pembrolizumab cohort (hazard ratio 0.80, with a 1-year survival rate of 37% versus 26%). Both of these studies encompassed both HPV positive and HPV negative participants. An exploratory subgroup analysis of CHECKMATE-141 suggests that anti-PD-1 therapy may be more efficacious in HPV positive tumors (with a median overall survival of 9.1 months versus 4.4 months, as compared to a median overall survival of 7.5 months versus 5.8 months in HPV negative tumors); however, no such improvement was discerned in the HPV positive subgroup within the KEYNOTE-040 trial. The KEYNOTE-048 trial (23), a phase III randomized study comparing monotherapy with pembrolizumab, combination therapy with pembrolizumab and chemotherapy, and chemotherapy alone in the first-line setting of recurrent/metastatic HNSCC, indicates that pembrolizumab, either as monotherapy or in combination, confers superior outcomes over standard chemotherapy irrespective of HPV status. Collectively, these findings underscore the efficacy of anti-PD-1 agents in HNSCC, albeit the differential impact on HPV positive versus HPV negative tumors remains elusive. In comparison with standard chemotherapy regimens, the augmented sensitivity of both HPV(+) and HPV(-) HNSCC to immune checkpoint blockade may reflect a myriad of distinct mechanisms that foster inflammation and immunogenicity. HPV(+) HNSCC tumors typically exhibit a lower mutational burden; viral antigens serve as potent immunogens, prompting the infiltration of HPV antigen-specific CD8+ T cells. Conversely, HPV(-) HNSCC tumors present with a moderate to high mutational burden, potentially enriching neoantigen-specific CD8+ T cells (24). We did not observe that HPV status imparted a significant influence on the efficacy or prognosis of ICI, though the sample size of HPV(+) HNSCC patients was notably small, necessitating further large-scale studies to elucidate this issue.

The correlation between PD-L1 expression and the efficacy of ICI has been the subject of extensive analysis, yielding conflicting outcomes. Both Scheff et al. (25) and our own investigations failed to observe that a high CPS was predictive of enhanced ICI efficacy. However, in the Checkmate 141 trial (26), employing a threshold of ≥ 1% tumor membrane PD-L1 expression as the criterion for inclusion, nivolumab demonstrated a more pronounced reduction in mortality risk for PD-L1 positive patients compared to standard treatment, whereas PD-L1 negative patients exhibited a mortality risk ratio of 0.89 (95% CI: 0.54-1.45). The most recent analysis (27), conducted following an extended period of follow-up, reveals that the therapeutic benefits of nivolumab for PD-L1 negative patients augment over time, with the mortality risk ratio diminishing to 0.73, while the advantages for PD-L1 expressing patients remain consistent. When compared to the solitary expression of tumor PD-L1 in HNSCC, the incorporation of PD-L1 expression within tumor infiltrating lymphocytes (TIL) yielded a superior predictive efficacy. For instance, a retrospective analysis of patients undergoing pembrolizumab treatment indicated no significant variation in response based on tumor PD-L1 expression alone (defined as ≥ 1%). However, when both tumor and TIL PD-L1 expression were considered in tandem, PD-L1 positive HNSCC patients exhibited a significantly enhanced response rate, PFS, and OS (28).

Limitations of the current study must be acknowledged. First, our sample size was relatively small, it might decrease our statistic power. Second, this investigation is grounded in a highly heterogeneous cohort of patients, characterized by diverse primary tumor localizations. Third, external validation of a large cohort via multicenter studies is warranted before clinical application. Fourth, definition of allergy history was mainly based on self-report, future study should consider objective measures, such as lgE level assessment and skin prick test.

In conclusion, patients exhibiting a clinically beneficial response to immunotherapy were likely to demonstrate a history of allergies, but survival analysis suggested that a history of allergies did not confer a protective effect against disease progression and mortality in patients undergoing ICI treatment for recurrent or metastatic HNSCC. The findings of this research underscore the potential utility of allergy history in predicting immune therapy responses and identifying candidates particularly well-suited for immunotherapy. Additional confirmatory investigations are imperative to delineate the intrinsic mechanism underlying this correlation, including the examination of the functions of Th9 cells, interleukin-9, antihistamines, and other pertinent factors.

## Data Availability

The original contributions presented in the study are included in the article/supplementary material. Further inquiries can be directed to the corresponding author.
